# Associations between learning community engagement and burnout, quality of life, and empathy among medical students

**DOI:** 10.5116/ijme.5bef.e834

**Published:** 2018-11-30

**Authors:** Sean Tackett, Scott Wright, Jorie Colbert-Getz, Robert Shochet

**Affiliations:** 1 Johns Hopkins Bayview Medical Center, Baltimore, Maryland, USA; 2 University of Utah School of Medicine, Salt Lake City, Utah, USA

**Keywords:** Learning community, engagement, burnout, quality of life, empathy, medical students

## Abstract

**Objectives:**

To inform evidence-based design and implementation of medical school learning communities (LCs) by investigating which LC components medical students at one school with a multi-component LC were most valued and which were associated with desirable outcomes.

**Methods:**

In this cross-sectional study, all Johns Hopkins School of Medicine (JHSOM) students were surveyed in Spring 2016 regarding perceived value of LC components (peers, faculty advisors, Clinical Foundations of Medicine (CFM) clinical skills course, quarterly reflective discussion sessions, social activities, and LC rooms) with learning environment (LE) perceptions, quality of life, burnout, and empathy assessed as outcomes. Multivariate logistic regressions analyzed associations between LC components and outcomes.

**Results:**

Overall 368/480 (77%) students responded.  CFM was highly valued by 286 (80%) students, advisors by 277 (75%).  All LC components were significantly associated with favorable overall LE perceptions, but associations with LE subdomains varied. CFM was the only LC component to have significant associations with greater empathic concern (OR 2.1, 95% CI=1.2-3.7) and perspective-taking (OR 1.8, 95% CI=1.0-3.1), less emotional exhaustion (OR 0.4, 95% CI=0.2-0.6) and depersonalization (OR 0.3, 95% CI=0.1-0.5), and good quality of life (OR 3.7, 95% CI=1.9-7.1).  Every other LC component, except LC rooms, was associated with greater empathy or enhanced well-being.

**Conclusions:**

Components within an LC are valued differently and vary in their relationships with student outcomes.  Future LC research may isolate the effects of and explore interactions among different LC components, leading to more purposeful LC design and allocation of resources.

## Introduction

Learning communities (LCs) in undergraduate medical education can be defined as “longitudinal groups that aim to enhance students’ medical school experience and to maximize learning.”^1^  A growing body of evidence suggests that LCs can benefit medical students in a variety of ways, enhancing their perceptions of the learning environment,^2^^,^^3^ connections with peers and mentors,^4^^,^^5^ satisfaction with advising programs,^6–9^ performance in clerkships,^10^ and involvement in leadership and service activities.^3^  LCs can also benefit faculty participants by improving their clinical skills^11^ and job satisfaction.^12^  Perhaps as a result of these benefits, the number of US medical schools with LCs has increased dramatically, from 18 in 2006 to 102 in 2014.^13^ To date, most studies have treated LCs as single interventions. However, within one institution’s LC program, there is typically a variety of curricular, advising, and extracurricular activities.^1^^,^^14^ Across institutions, there is variation in how LCs are structured, supported, and oriented. For example, in 2012, a study of 66 LCs in the US and Canada found that LCs could have between 1 and 80 different student groups, involve between 1 and 125 faculty, cover 53 different curricular topics, and command a budget ranging from $10,000 to $1,400,000.^1^  At the heart of LCs are longitudinal relationships among students and between students and faculty, with pedagogic and social learning embedded within LCs in ways that deepen learning and create a sense of wholeness for students.^15^^,^^16^ However, there remains a dearth of understanding about how LCs should be structured to achieve their goals.  Because institutions may face different challenges and have different resources to devote to LCs, an understanding of how students value LC components and how they relate to relevant student outcomes could lead to more evidence-based LC design and implementation.

The goal of this study was to determine which LC components were most valued by students and most closely related to measurable outcomes at the Johns Hopkins School of Medicine (JHSOM), which has a mature LC that incorporates elements commonly used in medical schools.  As LCs appear to be associated with enhanced perceptions of the learning environment^2^^,^^3^ and aspire to enhance student well-being and empathy,^8^^,^^17^ we selected LE perceptions, empathy, quality of life, and burnout as our outcome variables.  Because LCs contain curricular, professional, relational, and social aspects, we hypothesized that LC components would be associated with student outcomes in different ways, but that in general, placing a higher value on LC components would be associated with more favorable LE perceptions, greater empathy, and better student well-being.

## Methods

### Study setting

The JHSOM Colleges Advisory Program is an LC that began in 2005 to enhance students’ clinical skills, professional formation, academic and career advising, and wellness.  The LC is organized into 4 “Colleges,” comprising students from all years of medical school.  Within each College are smaller groups called “Molecules,” made up of 5 students and their LC faculty advisor.  In their Molecules, students engage in the Clinical Foundations of Medicine (CFM) clinical skills course during their first year and have quarterly reflective discussions during all 4 years.  The LC also sponsors social activities, and each College is provided a designated space in the medical school building.  Table 1 provides additional details of these LC components.

### Study design and participants

Data for this cross-sectional study were collected as part of an annual survey, which was distributed electronically to all actively enrolled JHSOM students at the end of the 2015-2016 academic year.  We selected to survey all actively enrolled JHSOM students because all would be engaged in the LC at the time of the survey. The study received expedited approval from the JHSOM Institutional Review Board.

### Survey composition

#### Perceived value of LC components

Based on our previous research and experience leading LCs, we developed seven items that were intended to isolate discrete LC components that may already be in place or could be implemented at other institutions.  Items asked about the “value to you” for each LC component, with response options along with a five-point Likert scale (1= “No value”, 2= “A little value”, 3= “Some value”, 4= “A lot of value” 5 = “Exceptional value”).  The 7 LC components were (1) interacting with peers in Molecules, (2) interacting with peers in Colleges, (3) interacting with faculty advisors, (4) participating in the Clinical Foundations of Medicine (CFM) course, (5) participating in reflective discussion sessions, (6) participating in social activities (e.g. Olympics, happy hours), and (7) having a College room.

**Table 1:** Components of the Johns Hopkins School of Medicine learning community

Component	Definition
Peers in Colleges	Students are randomly assigned^*^ to one of four Colleges on matriculation and remain affiliated with their College through graduation.
Peers in Molecules	Within each College, students in each class year are randomly aggregated* into groups of five and assigned to a longitudinal faculty career advisor.
Advisor	Faculty advisors teach students in their Molecules weekly in CFM and quarterly for reflective discussions on professional growth. They meet students on their first day of medical school, meet individually 3 or more times each year, and participate with students in their transitions and milestones across the four years.
Clinical Foundations of Medicine (CFM)	Students and advisors spend 50 hours together in Molecules over 16 weeks during their first semester, learning patient-doctor communication, medical history-building, the physical exam, and professionalism.
Reflective discussion sessions	During intersessions that occur 4 times per year, advisors facilitate 90-minute reflective sessions with students in Molecules, focused on critical incidents and professional growth.
Colleges room	Each of the 4 Colleges has a dedicated multi-purpose suite with lockers, kitchen, social and study spaces on the second floor of the medical school building.
Social activities	The learning community program hosts school-wide events, such as an annual Olympics competition among Colleges.

*Balanced for gender and diversity in geographic, academic, and personal backgrounds

#### Learning environment perceptions

Learning environment (LE) perceptions were measured using the Johns Hopkins Learning Environment Scale (JHLES).^18^ The JHLES has 28 items, each with five-point response options. During development, exploratory factor analysis resulted in seven domains: (1) Community of Peers, (2) Faculty Relationships, (3) Academic Climate, (4) Meaningful Engagement, (5) Mentorship, (6) Inclusion and Safety and (7) Physical Space. Each item is scored 1-5 so that JHLES totals can range from 28 to 140, with higher scores indicating a more positive LE perception. Validity evidence for content, response process, internal structure and relationship to other variables for the interpretation of scores from the JHLES have been described in previous studies.^18–21^

#### Quality of life

Quality of life was assessed using a single-item linear analog self-assessment commonly used in other medical education studies^22^^,^^23^ and in a broad range of other quality of life research.^24–26^ This item asked individuals to rate their overall quality of life on a 1–5 scale, ranging from “as bad as it can be” to “as good as it can be.”

**Table 2:** Characteristics and baseline variables for 368 JHSOM students surveyed Spring 2016

Items		All	MS-1	MS-2	MS-3	MS-4	p value
		n (%)	n (%)	n (%)	n (%)	n (%)	
Demographics	Respondents	368 (100)	98 (26.6)	93 (25.3)	89 (24.2)	88 (23.9)	
	Age in years	26.0 (4.7)	24.1 (1.9)	26.2 (8.2)	26.4 (2.5)	27.4 (2.4)	<.001
	Male	192 (53)	48 (49)	56 (61)	49 (56)	39 (45)	0.227
Burnout	Emotional exhaustion	142 (38.6)	42 (42.9)	31 (33.3)	48 (53.9)	21 (23.9)	<.001
	Depersonalization	75 (20.4)	18 (18.3)	15 (16.1)	27 (30.3)	15 (17.0)	0.062
Quality of life	Good quality of life	286 (77.7)	76 (77.6)	74 (79.6)	59 (66.3)	77 (87.5)	0.008
		mean (SD)	mean (SD)	mean (SD)	mean (SD)	mean (SD)	p value
JHLES	Peers	3.6 (0.9)	3.6 (0.9)	3.6 (0.9)	3.4 (0.9)	3.9 (1.0)	0.001
	Faculty	4.0 (0.7)	3.9 (0.6)	4.1 (0.6)	4.0 (0.8)	4.2 (0.7)	0.048
	Academic	3.6 (0.7)	3.4 (0.7)	3.7 (0.7)	3.5 (0.8)	3.9 (0.7)	<.001
	Engagement	3.7 (0.8)	3.3 (0.8)	3.8 (0.7)	3.5 (0.8)	4.0 (0.7)	<.001
	Mentorship	3.9 (0.9)	3.9 (0.8)	3.7 (0.9)	3.9 (1.0)	4.3 (0.8)	0.001
	Safety	3.6 (0.8)	3.6 (0.8)	3.6 (0.9)	3.6 (0.8)	3.7 (0.8)	0.719
	Space	4.1 (0.7)	4.0 (0.7)	4.2 (0.7)	4.0 (0.6)	4.2 (0.6)	0.063
	Total	3.8 (0.6)	3.7 (0.5)	3.8 (0.6)	3.7 (0.6)	4.0 (0.6)	0.001
IRI	Empathic concern	28.7 (4.2)	28.7 (3.8)	28.9 (4.7)	28.9 (3.9)	28.3 (4.3)	0.790
	Perspective taking	27.9 (4.2)	27.6 (4.0)	28.0 (4.2)	28.1 (4.2)	27.9 (4.4)	0.846

Note: p values correspond to statistical testing by class year using ANOVA for tests of means and Chi2 for tests of proportions so that significant values indicate that there was a difference between values in at least two classes. MS = Medical Student, with numbers corresponding to a year in the 4-year medical school curriculum they were completed at the time of the survey JHLES = Johns Hopkins Learning Environment Scale means correspond to a 1-5 Likert scale for each item IRI = Interpersonal Reactivity Index, a measure of empathy. Means are totals for 7 item subscales scored 1-5 for each item. The range could be 7-35.

**Figure 1:**
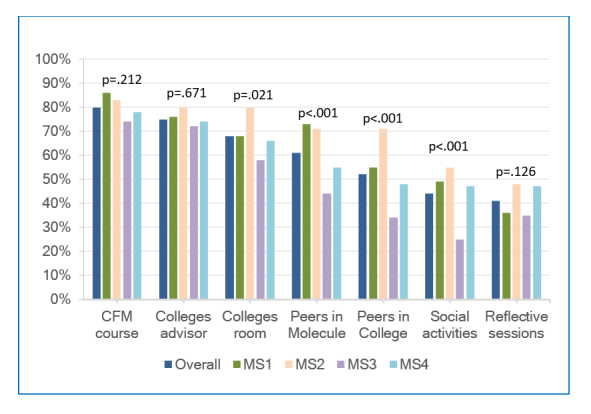
Percentages of students rating each LC component as having “exceptional” or “a lot” of value. Note: p values correspond to ANOVA tests by class year so that significant values indicate that there was a difference between values in at least two classes. CFM = Clinical Foundations of Medicine

#### Burnout

Burnout was assessed using two single-item questions validated in previous studies,^27–29^ asking respondents to report along with a seven-point Likert scale (1 = daily, 7 = never) how often they felt “burned out from my work” (for emotional exhaustion) or “callous toward people” (for depersonalization).

#### Empathy

Empathy was assessed using the Interpersonal Reactivity Index (IRI),^30^ which has been widely used in the general population.  The original instrument included four subscales: (perspective-taking, empathic concern, fantasy, and personal distress).  We included items for perspective-taking (which measures the cognitive domain of empathy) and empathic concern (which measures the affective domain of empathy).  These 2 subscales correlate with the Jefferson Scale of Empathy^31^ and have been used previously to measure empathy in medical students.^32^ Each subscale consists of 7 items, scored 1-5 along a Likert scale, with higher scores indicating greater empathy.

**Table 3:** Adjusted odds ratios for valuing learning community components and having favorable perceptions of the learning environment

LC component	JHLES Total	JHLES domains						
		Peers	Faculty	Academic	Engagement	Mentorship	Safety	Space
CFM course	3.9^***^	2.7^***^	4.0^***^	3.1^***^	2.9^***^	2.4^**^	2.1^**^	6.1^***^
Colleges advisor	4.1^***^	2.1^**^	5.3^***^	2.9^***^	2.2^**^	1.7	1.5	3.0^**^
Colleges room	2.2^**^	2.8^***^	1.7	1.6	2.5^**^	1.0	1.3	2.9^**^
Peers in Molecule	3.7^***^	3.8^***^	2.7^**^	2.0^**^	2.5^***^	1.6	1.1	3.3^**^
Peers in College	2.7^***^	3.1^***^	2.3^**^	2.0^**^	3.1^***^	1.4	1.4	2.4^*^
Social activities	4.4^***^	4.1^***^	2.9^**^	2.1^**^	3.7^***^	1.6	1.2	3.5^**^
Reflective sessions	2.7^***^	1.8^*^	3.3^**^	2.2^**^	2.3^**^	1.5	1.0	4.0^**^

*p<.05; **p<.01; ***p<.001

Odds ratios are adjusted for student sex and year in medical school. JHLES = Johns Hopkins Learning Environment Scale; CFM = Clinical Foundations of Medicine; JHLES total and domain ratings were dichotomized at item mean of 3.5; LC component item ratings were dichotomized as “exceptional” and “a lot” of value vs. other.

**Table 4:** Adjusted odds ratios for associations between LC components and quality of life, empathy, and burnout

LC component		IRI domain		Burnout domain	
	Quality of life	Empathic concern	Perspective taking	Emotional exhaustion	Depersonalization
CFM course	3.7^***^	2.1^*^	1.8^*^	0.4^***^	0.3^***^
Colleges advisor	1.3	2.6^**^	1.6	0.6^*^	0.5^*^
Colleges room	1.4	1.6	1.4	0.8	0.7
Peers in Molecule	2.7^**^	1.9^*^	1.4	0.9	0.7
Peers in College	2.4^**^	1.9^**^	1.5	0.9	0.6
Social activities	1.9^*^	1.0	0.9	0.8	0.9
Reflective sessions	1.7	2.5^***^	3.2^***^	0.9	0.7

*p<.05; **p<.01; ***p<.001

All odds ratios are adjusted for student sex and year in medical school and total JHLES score. CFM = Clinical Foundations of Medicine LC = Learning Community; IRI = Interpersonal Reactivity Index, a measure of empathy; LC component “value to you” dichotomized as “exceptional” and “a lot” of value vs. other; IRI domains were dichotomized at item mean of 4 on a 1-5 Likert scale; Burnout domains were dichotomized as high vs. not high; Quality of life was dichotomized as good or excellent vs. other.

### Data analysis

Basic descriptive statistics were tabulated, with ANOVA and Chi-squared tests for significant differences across class years applied as appropriate.

In bivariate and multivariate analyses, for ease of data interpretation, we dichotomized variables to create odds ratios in logistic regression models.  Sensitivity analyses showed that the strengths of associations were not affected when variables were dichotomized compared to if they were treated as ordinal or continuous variables.  LC component value was dichotomized by aggregating “exceptional” and “a lot” of value vs. other.  JHLES total and domain scores were dichotomized at item means of 3.5, which corresponded to more favorable than unfavorable ratings and approximated the median in most cases. Quality of life was dichotomized as good (aggregating “as good as it can be” and “somewhat good”) or not good. Burnout was dichotomized as high (weekly or more often)^27–29^ or not high. Empathy was dichotomized at item mean of 4, which approximated the sample median.  In logistic regression models, we analyzed the association of highly valuing each LC component with favorable JHLES total and domain ratings.  Based on previous work, we hypothesized that effects of LCs on wellness could be mediated through their LE perception.^1^^,^^20^^,^^33^ Accordingly, we adjusted for overall JHLES score in multivariate logistic regressions using empathy, burnout, and quality of life as dependent variables.  All models adjusted for student gender and year in medical school. Stata 13 was used for all data analyses.

## Results

Overall 368/480 (77%) students responded to our survey with response rates across each class exceeding 70%. The average age was 26 years (SD 4.7), and 192 (53%) students were male (Table 2).

Statistically significant differences were found across class years for overall JHLES score (F_(3,364)_ = 7.38, p=0.0001) and its domains of “Community of Peers” (F_(3,364)_ = 5.36, p=0.0013), “Academic Climate” (F_(3,364)_ = 7.97, p=<0.0001), “Meaningful Engagement” (F_(3,364)_ = 12.26, p=<0.0001), and “Mentorship” (F_(3,364)_ = 7.32, p=0.0001). Differences were also seen across classes for emotional exhaustion (χ^2^_(3, N = 368) _= 18.7, p<0.001) and quality of life (χ^2^_(3, N = 368) _= 11.8, p=0.008).  IRI measures of empathy were similar across all class years (Table 2).

### Perceived value of LC components

Overall, 286 (80%) students placed high value on participation in the Year 1 CFM course, and this high value assessment was found even among 69 (78%) fourth year students.  A total of 277 (75%) placed high value on interacting with their advisors.  The strongest differences across classes were found for interacting with peers in Molecules (χ^2^_(3, N = 368)_ = 22.9, p<0.001), interacting with peers in Colleges (χ^2^_(3, N = 368)_ = 26.4, p<0.001), and participating in social activities (χ^2 ^_(3, N = 368)_ = 19.1, p<0.001) (Figure 1).

### LC components and learning environment (LE) perceptions

All LC components were significantly associated with overall JHLES score and multiple LE domains.  After adjusting for gender and class year, the largest magnitudes of associations between LC components and JHLES total were seen with participation in social activities (OR 4.4, 95% CI=2.5-7.8), interacting with advisors (OR 4.1, 95% CI=2.4-6.9), and participation in the CFM course (OR 3.9, 95% CI=2.2-7.0) (Table 3).  The CFM course had strongly significant associations (p<.01) with all 7 JHLES domains.  The JHLES “Meaningful Engagement” domain was associated with every LC component at p<.01, while the “Mentorship” and “Inclusion and Safety” domains had no statistically significant associations with LC components (other than the CFM course).

### LC components and quality of life, empathy, and burnout

After adjusting for effect of overall LE perception, the CFM course was the only LC component to have significant associations with greater empathic concern (OR 2.1, 95% CI=1.2-3.7) and perspective-taking (OR 1.8, 95% CI=1.0-3.1), less emotional exhaustion (OR 0.4, 95% CI=0.2-0.6) and depersonalization (OR 0.3, 95% CI=0.1-0.5), and a good quality of life (OR 3.7, 95% CI=1.9-7.1).  Interactions with one’s advisor were significantly associated with empathic concern and less burnout, while reflective sessions were significantly associated with both empathic concern and perspective taking (all p<0.05).  Interactions with peers in one’s Molecule and one’s College were significantly associated with quality of life and empathic concern.  Valuing the College room was not associated with any of these outcomes (Table 4).

## Discussion

In this study of 368 JHSOM medical students, we found variation in how students valued learning community (LC) components and in how valuing LC components related to learning environment (LE) perceptions, quality of life, burnout, and empathy.  The Clinical Foundations of Medicine (CFM) course was unique among LC components for being highly valued by large majorities of students across class years and for having positive associations with each outcome we measured.

LCs are meant to enhance students’ learning experiences, which should in turn improve LE perceptions.  Rosenbaum described improvements in students’ LE perceptions at the University of Iowa after LC implementation.^3^  Similarly, Smith found that students at 16 medical schools with LCs had more positive perceptions of the pre-clerkship LE as compared to students at 8 schools without a LC, with significant differences for nearly every item on the Medical School Learning Environment Survey.^2^  In our study, all components of the JHSOM LC were related to positive overall LE perceptions, with specific LE domains from JHLES having different relationships with each LC component.  In particular, we found that “Meaningful Engagement”, which relates to a student’s sense that he or she is valued by the medical school, was linked with all 7 LC components we studied.  This could indicate that LCs that employ different combinations of LC components may improve student affiliation with their institution, something that has been receiving greater attention within the academic community.^34^^,^^35^  Conversely, the LE domains of “Inclusion and Safety”, which refers to students’ perceptions of discrimination and mistreatment, and “Mentorship”, pertaining to students’ perceptions of identifying clinical and research mentors other than their assigned LC advisors, were not closely related to LC components and therefore may not be as responsive to LC-based interventions.

Just as there were distinctive patterns in how LC components related to LE perceptions, there were variations in how they related to students’ empathy and well-being.  Valuing reflective sessions had a strong association with a single outcome, empathy, but no association with burnout or quality of life.  We cannot determine causation, but it is possible that these sessions - which were intended to provide students the opportunity to process their feelings on difficult encounters with a group of trusted individuals and thereby enhance empathy - may have had their intended effect.  Interactions with peers and advisors exhibited a second distinct pattern of relationships with outcomes, showing positive associations with more than one type, as each was associated with affective empathy and aspects of well-being.  This is consistent with previous work^20,36–38^ and suggests that structures that foster relationships may have broader impacts than others.  The Colleges rooms, which give groups of students specific places to congregate, was associated with yet another pattern, not having associations with any of the outcomes, despite being highly valued by over two-thirds of students.  While the physical environment and actual spaces are believed to influence learning and morale in medical education,^39^ how one creates settings that facilitate the enhancement of medical student connectedness and fulfillment is poorly understood. This may deserve further investigation considering the finances associated with building and renovations.

The CFM course was unique among LC components for being highly valued across all class years and having strong associations with favorable LE perceptions, greater empathy, and better well-being.  Clinical skills courses are common components of medical school LCs,^1^ and studies have consistently shown that early clinical experiences by medical students can have a variety of benefits, including fostering clinical skills,  stimulating professional development, sparking motivation, and bolstering confidence.^40^^,^^41^ Early clinical skills courses embedded within LC structures have likewise been associated with improved clinical evaluations.^10^^,^^42^ To our knowledge, ours is the first study to show associations between an LC clinical skills course and greater learner empathy and well-being.  Additionally, although the CFM course was conducted within peer Molecules and taught by the LC advisor, CFM was valued more highly and was more strongly associated with favorable outcomes than either of these individual LC components.  Likewise, while the timing of the course, as medical students are beginning their professional development in a new academic environment, could be significant, students are also engaged in sponsored social activities then, which we found to have few relationships with empathy and well-being. Our suspicion, which would need confirmation in future study, is that the unique structure and process of LC learning – small groups of students connected to a teacher who knows them beyond the boundaries of the classroom, journeying together over time, and learning with and from peers in this context – creates a “safe space” for learning and reflection that is greater than the sum of its parts, and may have an enduring impact on students’ professional development. ^43^ ^-^ ^45^

Several limitations of this study should be considered.  First, we can only describe associations among variables in a cross-sectional study; a longitudinal study may be able to demonstrate whether LC components cause improvements in student well-being and empathy. Second, although JHSOM’s LC model of curricular and advising components are shared by other medical school LCs,^46^a multi-institutional study would be needed to demonstrate whether our findings would be comparable at other schools with similar or different LC structures. Third, we used the students’ perceived value of the components of the LC as a marker for their engagement with each component based on their responses to items newly created for this study. While our process for creating the items generated content validity evidence and items’ use in this study generated relations to other variables evidence, future work would be needed to validate these items better. Finally, our surveys relied on student self-report which could be prone to bias.  Other measures, such as more objective measures of academic achievement related to CFM course competencies or a qualitative analysis of the LC experience would add to our understanding of the value of learning in an LC context.

## Conclusions

In conclusion, our study showed that LC components are valued differently by students and likely impact students in variable ways, suggesting that future LC research should seek to disentangle the effects that each LC component has.  By describing the associations LC components have with desirable student outcomes, we contribute evidence that could lead to more efficient resource allocation for those seeking to develop or refine their own LCs.  A significant finding in this study was the importance of the first-year LC clinical skills course, which suggests that LC educational structures interweaving curricular learning of clinical content, advising, and longitudinal peer and faculty relationships may be particularly beneficial.  We hope that this work can pave the way for future studies to understand better the role that LCs can play in medical students’ well-being, skill development, and professional formation.

### Conflict of Interest

The authors declare that they have no conflict of interest.
